# A Comprehensive Study of Therapeutic Applications of Chamomile

**DOI:** 10.3390/ph15101284

**Published:** 2022-10-19

**Authors:** Amit Sah, Punnoth Poonkuzhi Naseef, Mohammed S. Kuruniyan, Gaurav K. Jain, Foziyah Zakir, Geeta Aggarwal

**Affiliations:** 1 Department of Pharmaceutics, Delhi Institute of Pharmaceutical Sciences and Research, Delhi Pharmaceutical Sciences and Research University, Sector-3, M.B. Road, Pushp Vihar, New Delhi 110017, India; 2 Department of Pharmaceutics, Moulana College of Pharmacy, Perinthalmanna 679321, India; 3 Department of Dental Technology, College of Applied Medical Sciences, King Khalid University, Abha 61421, Saudi Arabia; 4 Center for Advanced Formulation Development, Delhi Pharmaceutical Sciences and Research University, Sector-3, M.B. Road, Pushp Vihar, New Delhi 110017, India; 5 Department of B. Pharm (Ayurveda), School of Pharmaceutical Sciences, Delhi Pharmaceutical Sciences and Research University, Sector-3, M.B. Road, Pushp Vihar, New Delhi 110017, India; 6 Department of Pharmaceutics, School of Pharmaceutical Sciences, Delhi Pharmaceutical Sciences and Research University, Sector-3, M.B. Road, Pushp Vihar, New Delhi 110017, India

**Keywords:** apigenin, bisabalool, chamomile, chamomilla, flavonoid, matricaria

## Abstract

Chamomile has a long history of traditional medicinal uses. The two commonly used varieties with therapeutic applications are German chamomile known as *Matricaria chamomilla* L. and Roman chamomile or *Chamaemelum nobile* L. The plant contains many components, namely, flavonoids, terpenoids, and coumarins, which are responsible for its medicinal properties. The review discusses recent developments that help in establishing its role as a therapeutic agent in various areas as an anti-inflammatory, antioxidant, analgesic, antimicrobial, hepatoprotective, anti-allergic, anticancer, and anti-hypertensive agent. Not much is known about its role in the treatment of CNS disorders and metabolic syndromes, which are also discussed. The chemical components responsible for the therapeutic activity and the respective mechanism of action are also elaborated.

## 1. Introduction

The word “chamomile” comes from two Greek words, Chemos and Melos, meaning “ground apple” for its apple-like smell [1]. There are many varieties of Chamomile, and they are known by an array of names, such as Babuna camomile, German chamomile, Roman chamomile, English chamomile, Hungarian chamomile, Single chamomile, Camomilla, Flos chamomile, pinheads, sweet false chamomile, and scented mayweed. Chamomile is found everywhere, and it is a well-documented plant in the world [2]. Chamomile (*Matricaria chamomilla* L.) belonging to family Asteraceae (formerly Compositae) is an essential medicinal herb indigenous to Europe and Asia [3]. It is grown in southern and eastern Europe, northern Africa, central and western Asia and western North America [4]. Hungary is the main producer of the plant’s biomass. Mughal introduced this herb to India, and it is grown in the northern part of India. Seeds require open soil to survive so it often grows near roads and around landfills and cultivated fields as a weed. There are several botanical species known as chamomile, such as German chamomile, wild chamomile (*Matricaria discoidea* DC.), Valley mayweed (*Matricaria occidentalis* G.), *Matricaria aurea* Loefl., field or corn chamomile (*Anthemis arvensis* L.), stinking chamomile (*Anthemis cotula* L.), scentless or false chamomile (*Tripleurospermum inodorum* L.), dyer’s chamomile (*Cota tinctoria* L.), etc. [5]. To avoid confusions, *Matricaria recutita* L. (*Matricaria chamomilla* or *Chamomilla recutita*) is now considered the botanical name for chamomile belonging to genus *Chamomilla* L. and family *Asteraceae*. A contrasting feature between true chamomile and other varieties is either a stinking smell or odorless smell, which is otherwise fragrant.

Two major species of chamomile widely used for health conditions are German chamomile (*M. chamomilla* L.) and Roman or English chamomile (*Chamaemelum nobile* syn. *Anthemis nobilis* L.) [6]. There is a third species that is used commonly in cosmetics and perfumery industries, namely *Ormenis multicaulis* Braun-Blanq. & Maire, also known as Moroccan chamomile. *M. chamomilla* L. have more biological effects than other species [7]. In Europe, it is considered as a “cure all”, and in German, it is referred as “alleszutraut”, meaning that it is capable of anything. Chamomile is generally safe for consumption and is consumed as tea or tonic. It is a component of several traditional, Unani, and homeopathic medicinal preparations [8]. It is primarily used for the treatment of mild skin irritation and to treat anxiety, inflammation, and spasm or as a sedative. As a drug, it is useful in flatulence, colic, hysteria, intermittent fever, depression, ulcer, and wound healing, etc. In 2000, USFDA decided that chamomile can be used as an active ingredient in over-the-counter (OTC) dietary supplements. In addition, for use in food products, German chamomile is categorized as generally regarded as safe (GRAS). Furthermore, USFDA also recognizes essential oil, extracts, and distillates as GRAS. This stresses the importance of chamomile in the food industry. Nutraceuticals play an important role in the prevention of diseases. The possibility of its use as a nutraceutical agent can be explored if the phytocomponent is identified and explored in such a way that it provides high bioavailability as well as efficacy.

The pharmacopoeia of 26 countries included chamomile as a drug [6]. With the current knowledge of chamomile in the treatment of various diseases, its role as a pharmaceutical agent cannot be ignored. The progression of science has increased its knowledge on therapeutic benefits manyfold, making it a herb of pharmacological importance.

The review discusses recent invitro and invivo findings that elaborate the role of chamomile as an anti-inflammatory, antioxidant, analgesic, antimicrobial, hepatoprotective, anti-allergic, anticancer, and anti-hypertensive agent. Not much is known about its effects in the treatment of central nervous system (CNS) disorders and metabolic syndrome, which are also discussed. The maximum therapeutic potential can be harnessed only if the mechanism of action is known. Therefore, the paper highlights the chemical component and the possible mechanism of action responsible for chamomile’s medicinal properties. This will help future researchers in developing a suitable delivery system so that the natural product can be used as a therapeutic tool.

## 2. Morphology

**Figure 1:**
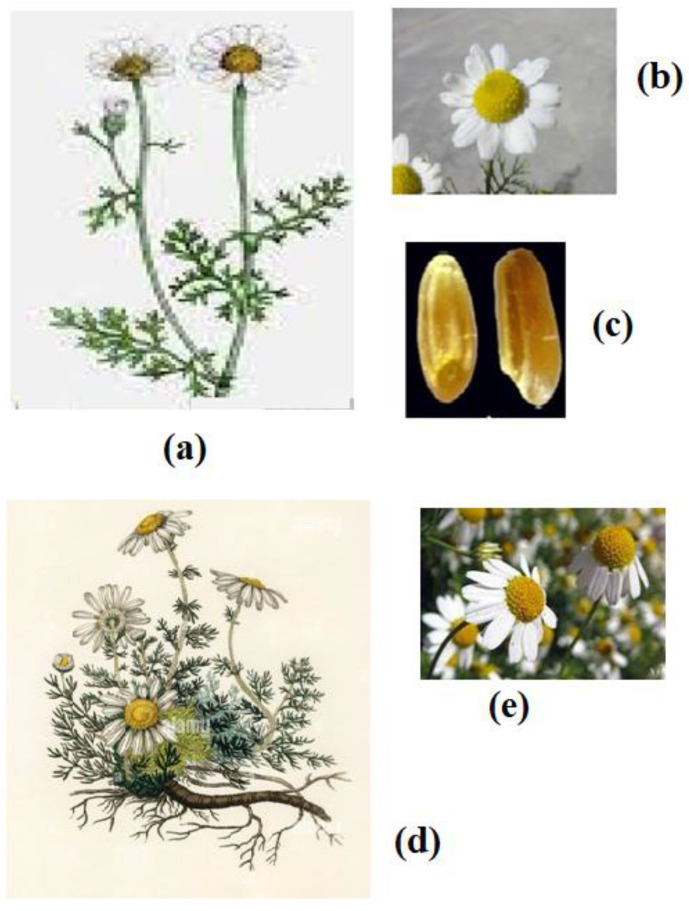
Morphology of German and Roman chamomile. The figure shows German chamomile plant (**a**), its flower head (**b**), seeds (**c**), Roman chamomile plant (**d**), and its flower head (**e**).

*M. chamomilla*: *M. chamomile* or *Matricaria recutita* L. or *Chamomilla recutita* L. is also known as German chamomile. It is an annual aromatic herb that carries a height of 10–60 cm (Figure 1a). It has feathery foliage with daisy-like white flowers and grows about 20 inches. The flowers have fragrance, but its foliage does not have any scents [9]. Flowers are arranged in heads or a capitulum as the outer ring ray and inner disc florets, a common characteristic feature of family *Asteraceae* (Figure 1b) [7]. The fruits produced are called achenes, which are cylindrical, 0.8–1 mm long, and around 0.5 mm wide, with three abaxial and two nearly marginal thin ribs (Figure 1c).

*C. nobile* L.: It is a perennial form, also known as Roman or English chamomile. It grows only one foot and their flowers have a scented smell. It is used as ground cover since it grows only 4–12 inches in height (Figure 1d). The foliage is feathery with an apple scent [10]. Its flowers are white in color anddaisy-like with down-turned petals (Figure 1e) [11]. The flowers are larger than German chamomile flowers.

The true chamomile is often confused with the plants of genra *Anthemis* due to the similar flower head [6].

## 3. Chemical Constituents

Biological applications of chamomile are related to its chemical components. The active constituents are mainly present in fresh or dried flower; therefore, infusions or essential oils are used in medicinal preparations. The flower yields a maximum of 2% of volatile oil, which houses more than 120 constituents. The main constituents of the oil include terpenoids, mainly sesquiterpenes and α-bisabolol [12]. The components present in essential oil, chamazulene, α-bisabolol, and cis-β-farnesene, are hydrophobic in nature. Chamazulene is not naturally present but proazulene and matricin present in chamomile flower heads are known to degrade into chamazulene during steam distillation processes. Other components such as flavonoids, coumarins, and phenolic acids are water soluble and, hence, exert therapeutic effects when chamomile is consumed as tea [13]. The major flavonoids present are apigenin, quercetin, patuletin, and luteolin in concentrations of 16.8%, 9.9%, 6.5%, and 1.9%, respectively, of course again depending on the species and cultivation. Approximately, 28 terpenoids and 36 flavonoids have been isolated from different varieties of chamomile. The coumarins present are herniarin and umbelliferone in 0.1% concentration. Mulinacci et al. [14] investigated the presence of 39% cinnamic acid derivatives such as ferulic acid and caffeic acid. An author claimed that leaves also house chemical components such as terpenoids, phenolic compounds, flavonoids, tannins, and phytosterols [15].

However, the chemical composition of the plant varies from place to place due to differences in the type of soil, the specific environment, and different genotypic backgrounds over different locations [8]. There is a marked difference in the chemical composition of German and Roman chamomile varieties (Table 1). The main components in German chamomile are terpenoid; α-bisabolol and its oxide azulenes, such as chamazulene (1–15%); and apigenin [12,16,17,18]. Roman chamomile, on the other hand, contains mainly angelic acid and tiglic acid esters [19].The essential oil obtained from German chamomile is deep blue in color due to high levels of chamazulene. Roman chamomile, on the other hand, produces light-blue essential oil, which turns yellow during storage due to oxidation. The chamazulene content in Roman chamomile essential oil is 5%, whereas it is 50% in German chamomile oil.

Apigenin is the main bioactive component and is, therefore, considered a quality marker of chamomile. European Pharmacopoeia suggests that chamomile flowers should contain at least 0.25% of apigenin-7-glucoside so that it can be used as a therapeutic agent. Similarly, the US pharmacopoeia states that dried chamomile flowers should contain no less than 0.3% in apigenin-7-glucoside and no less than 0.15% in bisabolan derivatives. It can be observed in Table 1 that apigenin and sesquiterpene contents are higher in German chamomile; therefore, flower extracts of this variety are generally used for biological applications.

Due to the increasing demand for German chamomile, its mass cultivation has already started. However, due to different environmental conditions, high essential-oil content is a challenge. Scientists have extensively studied the genetic diversity of German chamomile [4]. Genetic markers associated with α-bisabalool and chamazulene, have been identified, which can be incorporated during breeding. Furthermore, gene-transfer technology helped in the overexpression of sesquiterpene synthase, resulting in high sesquiterpene content [22]. Similarly, genetic engineering has helped in the production of essential oil with high yields of terpenoids [23].

## 4. Biological Activities of Chamomile

### 4.1. Anti-Inflammatory Activity

The human body is constantly exposed to both internal and external stresses in our daily lives. These can be injuries or infections that lead to the damage of biological membranes. Inflammation occurs in response to these stresses and participates in various repair pathways. In a study by Lee et al. [24], the effects of topical application of fixed oil from German chamomile were studied in an atopic dermatitis animal model. It was seen that after a 4-week administration period, there was a significant reduction in serum IgE and IgG1 levels. In another study, a combination of equal parts of powdered *Commiphora molmol* Engl. ex Tschirch and *Coffea arabica* L. and *M. chamomilla* L. flower extracts was studied for the treatment of irritable bowel syndrome (IBS) [25]. For the study, inflammation was induced by the administration of lipolysaccharides (LPS) from *E. coli* in a concentration of 100 ng/mL through the activation of THP-1 macrophages, which led to the release of various pro-inflammatory cytokine signals such as interleukin 6 (IL-6) and tumor necrosis factor alpha (TNF-α), as well as the stimulation of intestinal epithelial cells and the further release of chemokines, interleukin 8 (IL-8), and monocyte chemoattractant protein-1 (MCP-1). The results clearly demonstrated that the combination strongly inhibited the release of cytokine and chemokine mediators. The IC_50_ value displayed by the combination of extracts, for the inhibition of TNF-α, was found to be 26 µg/mL, whereas 98 µg/mL was exhibited when chamomile extract was used alone. Similarly, the IC_50_ values for inhibition of IL-8 and MCP-1 for the extract combination was found to be 59 µg/mL and 54 µg/mL, respectively, compared to 268 µg/mL and 39 µg/mL, respectively, when used alone. The results suggest that chamomile flower extracts can be used for the alleviation of inflammation associated with IBS. Pharmacological screening has shown that apigenin, its glycosides, and ferulic acid might be responsible for anti-inflammatory activities. It is known that nitric oxide (NO) is also responsible for inflammation. The activated macrophages stimulate the expression of inducible nitric oxide synthase (iNOS) gene, which produces NO synthase. Another author studied the mechanism of action of dried flower extracts from chamomile on inflammatory disorders [26]. Macrophages were stimulated by the administration of LPS, and the production of NO was assessed both in the absence and presence of the aqueous chamomile extract. In the absence of the chamomile extract, NO production significantly increased by 30-fold, whereas after treatment with chamomile in doses ranging 5–40 µg/mL, the levels reduced by 53–83%. Here, again, the inflammatory effect was believed to be associated with the presence of apigenin and its glycosides. However, another study published the effect of a flavonoid, luteolin, present in chamomile for its anti-inflammatory effects [27]. Invivo studies were carried out on mice using carrageenan-induced inflammation and air pouch models. The results demonstrated significant dose-dependent increases in anti-inflammatory responses. Fleming et al. [28] investigated the possible effects of matricin and chamazulene in inflammation. Human endothelial cells were treated with LPS to induce the expression of adhesion molecule intercellular adhesion molecule 1 (ICAM-1). ICAM-1 is known to be associated with inflammatory responses (TNF-α and interferon γ; IFN-γ) in endothelial cells. After treatment, a dose-dependent reduction in ICAM-1 levels was witnessed with a maximum effect of 52.7 ± 3.3% with 75 µM of matricin, whereas minimal effects were detected for chamazulene. Some authors also reported anti-inflammatory effects of essential oil components, such as α-bisabalool, bisabolonoxid [29], and polyketides [30].

### 4.2. Antioxidant Activity

Antioxidant and anti-inflammatory activities are linked to each other. Free radicals are produced in cells/tissues during normal physiology and play important roles that are necessary for normal functioning. However, excessive production is also dreadful as it can cause oxidative stress and damage cells, lipids, and proteins. Simultaneously, oxidative stress induces the expression of cyclooxygenase (COX) and lipooxygenase (LOX), which further triggers the secretion of inflammatory mediators. Several studies have been conducted, which have proven antioxidant activity by chamomile. For instance, Wang et al. [31] demonstrated that apigenin strongly inhibited free radical production and associated damage in the H_2_O_2_-induced model. In another study, antioxidant effects of hydroalcoholic chamomile extracts were studied on H_2_O_2_-induced human adenocarcinoma HT29 cell lines [32]. The herb significantly reduced reactive oxygen species (ROS) levels, with the most prominent effect witnessed at a dose of 1000 mg/mL. The most well-known marker for oxidative stress is 8-iso-prostaglandin F2α (8-iso-PGF2α), which is derived from ROS peroxidation. A significant reduction in 8-iso-PGF2α clearly signified radical scavenging activity by chamomile. Additionally, a 50% reduction in prostaglandin E2 (PGE_2_) levels was observed, which is linked to the inhibition of COX activity. Furthermore, the author also tested the anti-inflammatory activity on LPS induced model. A significant reduction in TNF-α and IL-6 was observed, which suggests anti-inflammatory activities. This study signifies that chamomile has the potential to be used in colorectal cancer. Although these studies have not identified the chemical constituent responsible for antioxidant activity, Parham et al. [33] indicated that flavonoids may be responsible.

### 4.3. Anti-Allergic Activity

The presence of allergic diseases has been increasing worldwide. Mast cells are present in most organs and tissues, and its activation triggers the release of histamine; inflammatory mediators such as leukotrienes, prostaglandins, and proteases; and proinflammatory cytokines. In a study by Chandrashekhar et al. [34], allergy was induced by treatments with compound 48/80, a mast cell stimulator, and treated with standard drug, disodium chromoglycate (control),and a methanolic chamomile extract. Chamomile extracts in concentrations of 300 mg/kg showed the inhibition of mast cell degranulation by 73.3% compared to 67.75% by disodium chromoglycate. Histamine levels also significantly reduced in treated groups vs. the control group. NO levels in serum, peritoneal, and bronchoalveolar fluids were also tested, and a reduction in approximately three-fold was observed with standard treatments, whereas chamomile extracts at a dose of 300 mg/kg showed maximum reduction by five-fold [34]. Another author tested the efficacy of topical chamomile oil on 2,4-dinitrochlorobenzene-induced allergic dermatitis. With serum IgE and IgG1, histamine levels were significantly reduced after 4 weeks and 2 weeks of applying chamomile oil, respectively [24].

### 4.4. Anti-Microbial Activity

Studies suggested that chamomile contains α-bisabolol, which gives it anti-microbial properties [35]. It has shown activity against both Gram-positive and Gram-negative bacteria. The antibacterial potential was studied by Kazemian et al. [36]. Wounds were created by incision using a blade and subsequently infected with *Pseudomonas aeruginosa* strains. Chamomile and tetracycline ointments were then applied and the results were compared. It was found that the group treated with chamomile showed reduced wound healing times (5.3 days) compared to the antibiotic group (6.3 days). The treatment of any microbial infection often becomes very difficult due to biofilm formation. Biofilms are highly structured microbial cells that enclose themselves in a self-produced extracellular matrix. They are responsible for bacterial or fungal resistance, which is almost impossible to eradicate. Studies have also reported that chamomile possesses the ability to disrupt biofilms. An invitro study was carried out in which tissue specimens exhibiting multi-drug resistance against *P. aeruginosa* were collected and cultured in a soya broth medium. A diluted methanolic chamomile extract was used, and the minimum inhibitory concentration (MIC) and minimum bactericidal concentration (MBC) were determined by broth microdilution methods. A chamomile extract measuring 50 µL and 150 µL of bacterial inoculums were placed into 96-well microtiter plates and incubated at 37 °C for 24 h. The MIC and MBC were found to be 12.5–50 mg/mL and 25 mg/L, respectively. The biofilm inhibition assay was also carried out and chamomile extracts in the concentration range of 1.6 to 100 mg/mL showed an inhibition of the biofilm [37]. The plant constituents can also be used in synergism with synthetic drugs to enhance their antimicrobial property. A study was conducted to determine the MIC values of chamomile essential oil and extracts in hexane, diethyl ether, dichloromethane against *Staphylococcus aureus*, *Escherichia coli*, and *Candida albicans*, and the values were compared with antibiotics such as ampicillin, cefuroxime, tetracycline, fluconazole, and nystatin. Individual MICs exhibited by extracts were comparatively higher than standard antibiotics; however, when they were used in combination, they displayed a synergistic/additive effect. The effect was most prominent with tetracycline, and a fraction inhibitory concentration index of 0.26–0.37 and a four-fold decrease in MIC against both Gram-positive and Gram-negative bacteria were observed. Since the different fractions of chamomile extract were used in the study, it suggests that multiple components play together and exhibit antimicrobial properties [38]. In another study, the cytotoxic effect of chamomile oil was tested on human keratinocyte and an epithelial cell line [39]. Different concentrations of chamomile oil at 16 µg/mL, 32 µg/mL, 125 µg/mL, and 250 µg/mL were tested. It was found that the oil showed toxicity only at the highest concentration. Furthermore, the author also tested antibacterial activities against Gram-negative strains of *E. coli, P. aeruginosa,* *Klebsiella pneumonia*, and *Enterobacter aerogenes* and Gram-positive strains of *S. aureus*; with respect to methicillin-resistant and methicillin-susceptible properties, and *Enterococcus faecalis*. The MIC for all the strains were found to be >1000 µg/mL. Upon testing synergistic activities, the additive effect was found with amoxicillin and doxycycline against *S. aureus*, and a synergistic effect was observed with penicillin V against *P. aeruginosa*. Similarly, another study reported that chamomile acetone extracts showed improved antimicrobial activity against *S. aureus* and *C. albicans* compared to traditional antibiotics [40]. The zone of inhibition was observed to be 27 ± 0.145 mm and 18 ± 0.22 mm against *S. aureus* and *C. albicans*, respectively, at a concentration of 400 µg/mL. The invivo studies suggested that topical application of chamomile cream on infected mice showed a complete cure in 14 days, whereas topical nystatin application showed redness and inflammation even after 17 days.

Antiviral effects of chamomile were also studied in acyclovir-sensitive and acyclovir-resistant Herpes simplex virus strains. Chamomile essential oil showed strong antiviral activity against both strains, as proven by a plaque reduction of 96.6–99.9%. The IC_50_ value was also found to be 0.003% [41]. More recently, anti-COVID effects were also witnessed upon taking TaibUVID supplements comprising chamomile, honey, and *Nigella sativa*. During the study, it was observed that 70% participants did not contract SARS-CoV2 infections even after contact with COVID-19-positive patients. Furthermore, 30% of COVID-19-positive patients took supplements, and symptoms improved in 1–4 days, and the patients had a negative RT-PCR test, while for the other 70%, the condition improved after 10 days of treatment [42].

### 4.5. Analgesic Activity

In a study by Chaves et al. [43], effects of a crude fraction of chamomile were tested for analgesic activity by formalin tests. Chamomile was characterized for the presence of polysaccharides, arabinose, galactose, xylose, and uronic acid. A reduced nociception (by 96%) was observed upon using a 30 mg/kg dose compared to the control (10 mL/kg of saline solution), which demonstrated analgesic property.

### 4.6. Anti-Cancer Activity

It was first reported by Srivastava et al. [44] that chamomile extracts possess anticancer activities. These authors tested its effects on human prostate cancer cells. Both aqueous and methanolic extracts showed a dose-dependent reduction in cell viability ranging between 6 and 37%, although responses were more prominent for methanolic extracts. Furthermore, the mechanism of action was investigated and three-fold increased apoptosis was exhibited by methanolic extracts. The anti-proliferative effect was also studied, and IC_50_ values of 1650–4000 µg/mL and 165–300 µg/mL were noted for aqueous and methanolic extracts respectively [44]. The effect of the ethanolic extract was tested by another author for anti-proliferative activity against the human hepatoma cancer cell line. The IC_50_ value was found to be 300 µg/mL, and there is 2,2-diphenyl-1-picryl-hydrazyl-hydrate (DPPH) scavenging activities of 94% by 1.5 mg/mL. The determination of chemical components revealed high amounts of polyphenols and flavonoids.

A wide variety of clinical cancer models have been used for the investigation of anticancer activity of chamomile and has shown anticancer activity against breast cancer, lung cancer, ultraviolet B (UV-B) induced skin cancer, oral carcinogenesis, and colon cancer etc. A detailed study pointed out that apigenin is the main component responsible for anticancer activity through apoptosis, anti-proliferation, and autophagy [45]. Since oxidation is another reason for the progression of cancer, the free-radical-scavenging activity shown by chamomile is another possible reason for anticancer activity [46]. Furthermore, chronic inflammation is also associated with the pathogenesis of cancer, and the anti-inflammatory role exhibited by chamomile may be an additional reason for its anticancer effects.

### 4.7. Central-Nervous-System-Related Disorders

Chamomile tea has long been used for calmness and sleep disorders. Some authors reported that the sedative effect is due to a flavonoid, apigenin, found in chamomile [47]. Apigenin acts by binding to benzodiazepine receptors present in the brain. The sedation effects were tested by assessing the locomotor activity in mice against a diazepam control. A dose-dependent decrease in locomotion was observed, with maximum effects at a dose of 30 mg/kg of the chamomile crude fraction. Mice usually demonstrate anxiolytic activity by burying noxious stuff. The number of marbles buried was tested, and it was reduced drastically upon administering chamomile, suggesting anti-anxiety effects [43]. A recent study also suggested that chamomile has the potential to significantly alleviate anxiety in a zebrafish light–dark test model. During GC-MS analyses, it was found that the major components present were pentadecyl-3-methyl-2-butenoate, hexadecyl-3-methyl-2-butenoate, 1-piperidinol, and trans-1-ethyl-3-methyl-cyclopentane [48].

It is believed that neuropeptide substance P might be the reason for depression in humans. It acts on its receptor, neurokinin-1, triggering the release of various substances leading to the onset of depression. Furthermore, it is also reported that fluctuations in cortisol levels are associated with CNS disorders [49]. For instance, lower cortisol levels are found in generalized anxiety disorder, and higher levels are found in chronic stress. The study has also linked hypothalamic–pituitary–adrenocortical(HPA) axis activities to depression. Another study has shown that elevated levels of adrenocorticotropic (ACTH) levels are associated with stress and anxiety [50]. Chamomile extracts have been found to possess neurokinin-1 receptor antagonist activity [51]. Furthermore, the inhalation of chamomile oil vapors has been found to reduce ACTH levels caused by stress induced by ovariectomy in rats [52]. In addition, reports have suggested that flavonoid components in chamomile modulate central neurotransmitter activities, such as a reduction in serotonin, dopamine, and monoamine oxidase activity and elevates catecholamine production and noradrenalin activity [53].

Chamomile has been found to possess ingredients that play important roles in CNS diseases such as epilepsy and Alzheimer’s disease. In a study carried out by Hashemi and group, convulsions were induced by the administration of kainic acid [54]. Apigenin was then administered orally at 50 mg/kg for 6 days. The treatment significantly reduced the onset and severity of seizures. Immunohistochemical analyses showed that apigenin reduced neurodegeneration by increasing the number of living neurons in the hippocampus. The study also proved that apigenin restores memory deficits in epilepsy. Chronic oxidative stress is the reason for the stimulation of neurodegeneration. Chamomile, being a natural antioxidant, possesses the ability to scavenge free radicals and, thus, can be effective for the management of neurological disorders such as Alzheimer’s disease, Parkinson’s disease, and cerebral ischemia [55].

### 4.8. Anti-Hypertensive Activity

Chamomile extracts are known to possess anti-hypertensive effects [56]. The chamomile extract was fed orally at a dose of 200 mg/kg to normal rats. It was found that it significantly reduced systolic and diastolic blood pressure as well as heart rate. The same dose was then administered to high salt-sucrose-diet-induced hypertensive rats and again showed significant reduction in blood pressure (BP) and heart rate. Mechanistic investigations revealed that this was due to 38% of reduced angiotensin converting enzyme (ACE) activities. Furthermore, the results were compared with a standard anti-hypertensive agent, captopril, and chamomile was found to be superior. A detailed investigation was carried out by another scientist who explored the potential of apigenin as an anti-hypertensive agent [57]. Chronic inflammation and oxidative stress are involved with the development of hypertension, and hypertension induced cardiac hypertrophy. The infusion of apigenin at a dose of 20 µg/h for 4 weeks demonstrated a reduced BP and heart rate. It also significantly suppressed the levels of oxidative stress markers, ROS, and superoxide dismutase (SOD). The inflammatory cytokines, namely, IL-1β, TNF-α, and IL-6, were also reduced. The study proved that apigenin acts as an anti-hypertensive agent through the modulation of nicotinamide adenine dinucleotide phosphate hydrogen (NADPH) oxidase-dependent ROS generation and inflammatory mechanisms.

### 4.9. Hepatoprotectiveproperties

In order to prove hepatoprotective effects of chamomile, 1,2-dimethylhydrazine was administered to rats to induce toxicity to liver, following the monitorization of liver enzymes’ levels, including aspartate transaminase (AST) and alanine transaminase (ALT). Results revealed that the treatment with aqueous chamomile extracts reduced the levels of AST and ALT by 33–37%. Pretreatment with chamomile before inducing toxicity also had a protective effect. A detailed investigation suggested that chamomile acted by modulating COX-2 and iNOS pro-inflammatory enzymes [58]. Furthermore, the liver’s protective effects may also be due to antioxidant properties exhibited by chamomile through the modulation of SOD and glutathione peroxidase [59].

### 4.10. Protective Effects on Metabolic Syndrome

It is suggested that enhanced levels of ROS are associated with the production of adipocytes. An analysis of blood and adipose tissue revealed higher amounts of lipid peroxidation, protein, and DNA oxidation products. Chamomile constituents act as natural antioxidants; thus, they play a pivotal role in the management of metabolic disorders such as obesity. In addition, ethanolic extracts of chamomile protect the β cells of the pancreas against ROS in diabetes-induced rats. Aqueous extracts also suppresses fasting sugar levels and exhibit moderate anti-hyperglycemic effects. Anti-lipidemic properties have also been found in aqueous extracts as it reduced the serum cholesterol levels in hyperlipidemic rats [60]. In a study on Roman chamomile, six new octulosonic acid derivatives were isolated, and they were tested for their anti-inflammatory activities [61]. The compounds resulted in the inhibition of iNOS and ultimately suppressed ROS production. Inflammation is a risk factor in metabolic disorders such as obesity and cardiovascular complications. Therefore, further studies were conducted to determine their role in the induction of peroxisome proliferator-activated receptors α (PPARα), peroxisome proliferator-activated receptors γ (PPARγ), and liver X receptor (LXR). These are considered major players in the regulation of lipid and carbohydrate metabolism. The components showed a 1–2-fold induction in the activity of PPARα, PPARγ, and LXR. It also leads to a 50% inhibition of NF-κB activities, strongly pointing towards anti-inflammatory responses. Although German chamomile has not been analyzed for these compounds, it can be explored so that its activity in metabolic disorders can be elucidated.

### 4.11. Other Therapeutic Applications

Chamomile has found uses in other diseases as well. For instance, the essential oil of chamomile was tested for amoebicidal activity against *Acanthamoeba castellani* Neff [62]. The IC_50_ values obtained by essential oil were within 20.839 ± 2.015, which are comparatively higher than the standard antiamoebic drug, chlorhexidine (2.643 ± 0.55), suggesting that they were cytotoxic to amoebic cells but minimally to the murine macrophage cell line. The mechanism of action was also elucidated, and it was found that α-bisabalool present in chamomile essential oils induced the leakage of cellular contents by increasing the permeability of the plasma membrane and apoptosis. Similarly, its efficacy as an anti-leshmenial agent is also proved [63]. Another author tested the anti-pruritic effect of essential oil of chamomile [64]. The oral administration of essential oil showeddose-dependent reduced scratching in animals compared with fexofenadine controls (10 mg/kg). The most possible reason can be antihistamine effects exhibited by bisabolol oxide A, which are found during chemical analyses. Chamomile has also found applications in the treatment of premenstrual syndrome as its efficacy as an analgesic has already been discussed [65]. Additionally, the flavonoids present in chamomile are known to act as antispasmodic agents [66]. Nephroprotective effects were also studied, where its activity in acute kidney injury model was tested [67]. α-bisabalool attenuated the cell damage caused during kidney injuries. The antioxidant property might be responsible for nephroprotective effects. Some other biological applications of chamomile are discussed in Table 2.

**Figure 2:**
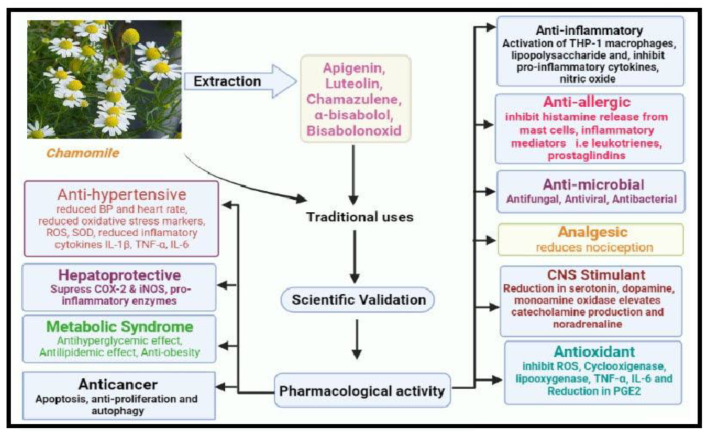
The therapeutic applications of chamomile and the respective mechanism of action.

Latest screening techniques such as LC-MS, GC-MS, etc., have made it possible to elucidate the active constituent responsible for pharmacological properties. Furthermore, it can be observed that the focus of most research studies is to establish the mechanism of action. This is very much necessary so that the natural product can be effectively utilized as a therapeutic tool. The possible mechanism of action of chamomile in different clinical uses is highlighted in Figure 2.

## 5. Conclusions

The identification of the phytoconstituent present in chamomile is important as it provides a chemical fingerprint of the extract so that qualitative and quantitative analyses can be performed. As we know, the processing condition of herbal materials affects the phytochemical composition; therefore, the identification of the chemical compound will help improve agricultural and processing technology. This will further help in the standardization of the herbal extracts and can prevent batch-to-batch variations. Moreover, with the latest developments that have helped us in providing detailed insights into the mechanism of action, it was possible to identify the biomarker responsible for biological activities. Once we know its physiological target, the phytochemical standard can be developed, which can help serve as a quality control for chamomile.

Most studies are limited to invitro/exvivo simulations. Extensive pre-clinical studies on animal models of various diseases are needed so that the effects can be extrapolated in clinical trials, which will help in establishing and validating chamomile as a therapeutic agent.

## 6. Future Prospects

The review highlights the biological activities exhibited by different chemical components present in chamomile. Many invitro studies have comprehensively detailed different therapeutic applications, suggesting chamomile as a promising herb. However, during the literature search, much evidence related to clinical use has not been found. It has been reported by many authors that chamomile, although a potentially useful herb, does not exhibit similar results during in vivo/clinical studies compared to invitro studies. There are many plausible explanations for this. For instance, chamomile essential oils contain lipophilic components thatare easily oxidizable, leading to degradation into oxides. Alcoholic and aqueous extracts of chamomile can be used, but then again, they display poor tissue permeability. Furthermore, the components are poorly soluble, which exhibit low bioavailability. In addition, the essential oil when used as a topical formulation or in inhalation therapy demonstrates irritation to the mucosa. Therefore, to enhance the safety and efficacy of chamomile, it is imperative to utilize concepts of novel delivery systems. The chamomile constituents can be encapsulated in lipid-based carriers such as nanoemulsions, nanocapsules, liposomes, etc., which will enhance its clinical acceptability and favourable application in medicine.

## Figures and Tables

**Table 1:** Chemical composition of German [20] vs. Roman chamomile [21]. The values represent average amounts for the two species. The content may vary depending on soil and climatic conditions.

Chemical Component	German Chamomile (% *w*/*w*)	Roman Chamomile (% *w*/*w*)
Esters	0.28	75
Aliphatic aldehydes	0.25	2
Ketones	0.5	3
Sesquiterpenes	35	3
Lactones and coumarins	9	2
Monoterpenes	1	5
Alcohols	20	5
Apigenin and its derivatives	0.39	0.12
Total flavonoid content	0.82	0.16

**Table 2:** Studies depicting the therapeutic activity of chamomile in various diseases.

Formulation	Activity	Disease	Study	Inference	Reference
Hydroalcoholic extract	Anti-inflammatory, antioxidant	Pulmonary fibrosis	Invitro	Different doses of chamomile extract were given (400, 600, 800, 1000, and 1500 mg/kg/day) to bleomycin-induced pulmonary fibrosis. 1500 mg/kg/day significantly reduced the damage to lungs.	[68]
Methanolic and aqueous extract	Antioxidant	Anti-helmintic	Invitro	IC_50_ values exhibited by methanolic and aqueous extracts against worms are 1.559 mg/mL and 2.559 mg/mL, respectively. The mortality rate of eggs shown by Albendazole standard was 91.75%, whereas, by chamomile extracts, it was 100% with a 0% recovery rate.	[63]
Topical nanoemulgel	Antioxidant, anti-inflammatory	Atopic dermatitis	In-vivo	The severity of lesions induced by capsaicin administration significantly reduced after the topical application of chamomile oil and nanogel. Furthermore, inflammatory markers, IL-4 and IL-22, and levels of nitric oxide also reduced considerably in the treatment group. Uponcomparison, it can be seen that nanoemulgel formulations showed better results than oil due to improved skin retention and penetration.	[69]
Nasal spray containing chamomile extracts	Antioxidant	Allergic rhinitis	Clinical trial	The antiallergy effects were evaluated on the basis of Sino-Nasal Outcome Test (SNOT) scores. A significant decrease in SNOT scores was observed in patients when treated with nasal sprays of isotonic seawater containing chamomile oil and steroid (mometasone furoate) compared to treatments comprising isotonic seawater with steroid, hypervolume seawater and steroid, and only steroid. Similar results were obtained for nasal mucociliary clearance time, which was significantly reduced.	[70]
Topical gel of chamomile alcoholic extract (3%)	Anti-inflammatory, analgesic	Chemotherapy induced oral mucositis	Clinical trial	The pain severity was assessed by numeric rating scale (NRS) every week for 21 days. Treatment with chamomile showed lower NRS scores compared to topical miconaz gel.	[71]
Oral mixture of linseed mucilage and chamomile flower decoction	Not known	Xerostomia	Clinical trial	The trial was conducted on 74 aged patients experiencing xerostomia. A proportion of participants (59.5%) felt the sensation of thick saliva at the end of the study period. The results were statistically significant (*p* < 0.05) compared to conventional saliva substitutes.	[72]
Oral chamomile oil and cumin oil	Antioxidant, hepatoprotective	Acetaminophen induced hepatotoxicity	In-vivo	The protective effects of cumin oil and chamomile oil were compared. Chamomile oil exhibited moderate protective effects as evident by decreased glutathione and superoxide dismutase activity in livers.	[73]
Chamomile extract in capsules	Anxiolytic effect	Generalized anxiety disorder (GAD)	Clinical trial	The effects were tested on 179 subjects with GAD, and Hamilton rating scalefor anxiety and depression was determined. All subjects showed antidepressant effects upon chamomile treatments, which suggests its possible role in anxiety and depression.	[53]
Chamomile oil drops	Analgesic	Cesarean Section Pain	Clinical trial	Pregnant women numbering 128 participated in the study. The subjects inhaled drops at 4, 8, and 12 h after surgery and the pain intensity was measured using visual analog scale. The findings indicated that chamomile oil significantly reduced the severity of pain in women compared to placebo drops.	[74]
Aqueous chamomile infusion	Estrogenic activity	Galactogogue	Case study	A 29-year-old woman started consuming chamomile infusion after the birth of a baby. After 3 months, milk production enhanced from 60 mL to 90 mL.	[75]
Alcoholic chamomile extract		Polycystic ovary syndrome	In-vitro	The production of cysts in the ovaries was stimulated by giving estradiol valerate injections. Subsequently, rats were treated with alcoholic chamomile extract (50 mg/kg) or corn oil only (control). It was seen that the cysts were reduced drastically after chamomile treatment. The levels of luteinizing hormone and follicle stimulating hormone also decreased considerably.	[76]

## Data Availability

Data sharing not applicable.
